# Hospital Sunday

**Published:** 1902-06-14

**Authors:** Joseph C. Dimsdale

**Affiliations:** Lord Mayor.


### THE INSTITUTIONAL WORKSHOP.

### HOSPITAL SUNDAY.

We have received from the Lord Mayor the following appeal:—

"The Mansion House, London, E.C.

"SIR,—Sunday next is the 30th anniversary of the institution of 'Hospital Sunday' in the Metropolis, and collections will then be made in every place of worship in the vast area of London in aid of the hospitals, dispensaries, and convalescent homes which do so much for the alleviation of the necessities of the suffering poor.

"The rapid increase in the population of London intensifies the need for these beneficent institutions, but unfortunately the subscriptions do not sufficiently keep pace with the requirements. Thus the largest and wealthiest city in the world, and the capital of the British Empire, labours under serious disadvantage in dealing with its sick population.

"To help to remedy this state of affairs it is necessary for at least £100,000 to be raised by next Sunday's offertories. Mr. George Herring, who has contributed large amounts in previous years, has now offered either to add one-fourth to the amount raised on Hospital Sunday by the various places of worship, or to give a donation of £10,000. The Council decided to accept the former offer, believing that it would stimulate other donors.

"With this munificent proposal, it is surely not hopeless to expect to raise the desired sum of £100,000.

"The Hospital Sunday Fund neither clashes nor competes with any other organisation, except in the friendliest rivalry, and it is hoped that this historic year may be rendered memorable in its annals by an unprecedentedly large collection in aid of his Majesty's suffering poor in London.

"Those who may be away from London or unable to attend their accustomed place of worship on Sunday are invited to send their contributions direct to me at the Mansion House.

"I am, Sir,

"Your obedient servant,

"JOSEPH C. DIMSDALE,

"Lord Mayor."

